# Breast Cancer Survivors’ Experiences With an Activity Tracker Integrated Into a Supervised Exercise Program: Qualitative Study

**DOI:** 10.2196/10820

**Published:** 2019-02-21

**Authors:** Hoi San Wu, Roxanne Gal, Niek C van Sleeuwen, Aarnout C Brombacher, Wijnand A IJsselsteijn, Anne M May, Evelyn M Monninkhof

**Affiliations:** 1 Human-Technology Interaction Group Department of Industrial Engineering & Innovation Sciences Eindhoven University of Technology Eindhoven Netherlands; 2 Julius Center of Health Sciences and Primary Care University Medical Center Utrecht, University of Utrecht Utrecht Netherlands; 3 Systematic Change Group Department of Industrial Design Eindhoven University of Technology Eindhoven Netherlands

**Keywords:** breast cancer, activity trackers, physical activity, sedentary behavior, qualitative research

## Abstract

**Background:**

There is growing evidence that physical activity is related to a better prognosis after a breast cancer diagnosis, whereas sedentary behavior is associated with worse outcomes. It is therefore important to stimulate physical activity and reduce sedentary time among patients with breast cancer. Activity trackers offer a new opportunity for interventions directed at stimulating physical activity behavior change.

**Objective:**

This study aimed to explore the experience of patients with breast cancer who used an activity tracker in addition to a supervised exercise intervention in the randomized UMBRELLA Fit trial.

**Methods:**

A total of 10 patients with breast cancer who completed cancer treatment participated in semistructured in-depth interviews about their experience with and suggestions for improvements for the Jawbone UP2 activity tracker.

**Results:**

The activity tracker motivated women to be physically active and created more awareness of their (sedentary) lifestyles. The women indicated that the automatically generated advice (received via the Jawbone UP app) lacked individualization and was not applicable to their personal situations (ie, having been treated for cancer). Furthermore, women felt that the daily step goal was one-dimensional, and they preferred to incorporate other physical activity goals. The activity tracker’s inability to measure strength exercises was a noted shortcoming. Finally, women valued personal feedback about the activity tracker from the physiotherapist.

**Conclusions:**

Wearing an activity tracker raised lifestyle awareness in patients with breast cancer. The women also reported additional needs not addressed by the system. Potential improvements include a more realistic total daily physical activity representation, personalized advice, and personalized goals.

## Introduction

Breast cancer is the most common cancer type among women worldwide [1]. In 2015, 14,551 new cases were diagnosed in the Netherlands. The overall 5-year survival rate is now 87%, and this rate is still increasing because of better treatment and earlier detection [2-4]. Breast cancer treatment comes with short- and long-term side effects. During and after treatment, patients often complain of fatigue, reduced fitness, and impaired quality of life [5].

A reduction in physical activity levels and increase in sedentary time is also seen as side effects [6,7]. However, previous exercise interventions have produced physical and psychological health benefits in patients with breast cancer, such as lower fatigue levels, increased physical fitness, and improved emotional well-being and quality of life [8]. In addition, evidence suggests that physically active patients with breast cancer have a lower risk of recurrence of the disease and mortality [9]. Thus, it is important to stimulate physical activity and reduce sedentary time among patients with breast cancer.

Physical activity trackers are a popular tool used in health interventions. They can stimulate people to be more physically active and less sedentary as they provide insights into physical activity patterns, resulting in greater feelings of empowerment to set and stick to health goals [10,11]. The use of activity trackers is in line with the motto of the Quantified Self movement [12]: “self-knowledge through numbers.” This movement reflects the fact that people increasingly integrate technology into their lives to gather personally relevant information. The underlying assumption about acquiring objective and quantitative data about one’s behavior is that it leads to more accurate self-knowledge, which in turn empowers users to improve themselves. A recent literature review demonstrated that there is mounting evidence for this “self-improvement hypothesis,” in which users gain usable insights from self-tracking data [13]. However, to date, few studies have used activity trackers in the rehabilitation of patients with cancer but are increasingly used for patients with cancer [14,15]. The first few studies explored the acceptability of activity trackers as a tool to stimulate physical activity and reduce sitting time and showed promising results [16-18]. However, more insight into the desires and needs of patients with breast cancer is needed to integrate an activity tracker in health care to increase physical activity and to achieve behavioral change.

The aim of this study was to explore experiences of patients with breast cancer with an activity tracker and its usage while participating in the intervention arm of the randomized controlled UMBRELLA Fit trial [19]. We used data collected from in-depth interviews to analyze the gap between the current and desired situations regarding the system’s information feedback and the user interface from the patient's point of view.

## Methods

### Participants and Intervention

This study is embedded in the UMBRELLA Fit trial [19] which investigates the effects of an exercise intervention on the short- (6 months) and long-term (24 months) quality of life of inactive patients with breast cancer after primary treatment completion. The exercise intervention is a 12-week program consisting of supervised sessions (strength and endurance training) at a physiotherapy center twice a week. In addition, patients are encouraged to develop an active lifestyle on the 5 other days, defined as being moderate to highly physically active for at least 30 min a day, and reduce sedentary time. To help them achieve these goals, patients developed personal physical activity goals in consultation with a physiotherapist, they kept an activity log, and the patients were given an activity tracker. The path to achieving these activity goals was discussed every 2 weeks with the physiotherapist and when needed, goals were adapted.

In total, 60 women with breast cancer completed the UMBRELLA Fit exercise intervention. At the time this qualitative study was performed, 13 women had completed the exercise intervention and were asked to participate in this qualitative study. Of these, 10 women agreed to this study and were approached by telephone. The women were at least 12 months post diagnosis and had completed their primary breast cancer treatment (except hormonal treatment). On an average, the women were interviewed 90 days after completion of the trial. The UMBRELLA Fit study was approved by the Medical Ethics Committee of the University Medical Center Utrecht (UMCU).

### The Activity Tracker

Initial requirements for the activity tracker were that it should possess the ability to track physical activity and inactivity, as well as the ability to synchronize this information to a database. In addition, the activity tracker should use an alert to remind the wearer of sedentary behavior that exceeded a certain amount of time.

The Jawbone UP2 (Jawbone, San Francisco, California, USA) met these requirements and was, therefore, used. This activity tracker is a wristband worn on the nondominant arm. It tracks steps, activity, and sleep and connects with a smartphone or tablet app. The Jawbone UP2 has been shown to have good reliability and validity in measuring the daily step count [20-22]. Using the accompanying “UP” app, users can monitor their step count, physical activity data, and calories burned. It is also possible to manually log workouts, track moods, and food intake, and to set certain goals for themselves. The UP app also incorporates a Smart Coach that provides personal, informative, and motivational messages and challenges based on what is measured. For example, *You are close to maintaining your 7-day, […] step average! Another […] steps, or a […] minute walk, will take you there,* and *Even if you don’t walk to work, you can still find moments to step. Hop off the bus one stop early. Park your car at the far end of the lot. It all adds up.*

In the UMBRELLA Fit study, the Jawbone UP2 was especially used to signal sedentary behavior by using the idle alert as an inactivity reminder: the Jawbone UP2 vibrated when the women were inactive for at least 45 min. Also, the daily step goal in the app was set at 10,000 steps as a guideline. The study did not focus on sleep, mood, or dietary intake.

Before the intervention, all women received the Jawbone UP2, downloaded the UP app on their smartphone if they had one, and set up an account. They were instructed to wear the Jawbone UP2 the whole day and to synchronize the activity tracker daily with the app.

### Interviews

The interviews were semistructured. We used a standard list of open questions in 5 categories to gain more insight into the users’ experiences with an activity tracker and its usage, added to a supervised exercise intervention (Textbox 1).

**Overview of the interview questions.**

1. Jawbone UP2 activity tracker
  - How did you experience the Jawbone UP2 (added value, shortcomings)?
  - Was it clear how to use the Jawbone UP2?
  - Did the Jawbone UP2 meet your expectations?
2. Idle alert
  - How was your experience with the idle alert?
  - Did it create awareness about your sedentary behavior?
  - What did you think of the 45-minute time span that triggered the idle alert?
3. UP app and Smart Coach
  - How was your experience with the UP app (added value, shortcomings)?
  - Was it clear how to use the app?
  - Which function(s) did you use?
  - Did the app contribute to creating awareness about your inactivity and activity?
4. Exercise intervention
  - What is your opinion about the addition of the Jawbone UP2 to the exercise intervention?
  - Did wearing the Jawbone UP2 activity tracker and using the UP app change your behavior?
5. Suggestions or remarks
  - Do you have any additional suggestions or remarks regarding the Jawbone UP2?

Face-to-face interviews were performed by 2 researchers (HSW and NCvS), with the exception of 1 patient who used the activity tracker but not the app and was interviewed by phone. The interviews were conducted at a location of the patients’ choice: in the hospital, in a lunchroom, or in their living room at home. All interviews were recorded using a smartphone. The interviews were transcribed verbatim and analyzed by 2 researchers (HSW and NCvS) following the guidelines of thematic analysis [23].

### Measures

Daily step count during the intervention period was measured with the activity tracker and synchronized to the app. After the intervention period, data from the activity tracker were downloaded from the Jawbone website. Information on usage of the activity tracker was obtained through logs in which women registered the daily wearing time. Before and after the exercise intervention, patients performed a maximal cardiopulmonary exercise test on a cycle ergometer to determine peak oxygen uptake (VO_2peak_). Finally, the level of physical activity was measured before and after the exercise intervention with the Short QUestionnaire to ASsess Health-enhancing physical activity (SQUASH) [24].

## Results

### Participants

After 10 interviews, data saturation was reached. The inter-rater reliability was calculated for 2 interviews using NVivo 11 (QRS International Pty Ltd, Melbourne, Australia). The Cohen kappa coefficient was 0.62, which is acceptable. The age of the women ranged from 33 to 64 years (median 57 [SD 8.8]).

### Adherence to Wearing the Activity Tracker

Jawbone UP2 data were available from 8 of the 10 women. Woman no. 7 stopped participating after 3 weeks because of dissatisfaction with different elements of the study, including the Smart Coach. Similarly, woman no. 8 had no smartphone and, therefore, data synchronization was not possible.

Overall adherence to wearing the activity tracker ranged from 35% to 99% of the days, with 5 of the 8 women having an adherence of 89% or more (Table 1). The 3 other women wore the activity tracker on 35%, 42%, and 67% of the days, respectively. The reasons for not wearing the activity tracker were problems with charging, the tracker broke down, holiday, and flu. Woman no. 6 wore the activity tracker for less than 10 days and did not register compliance consequently. She explained that she lost interest in the training sessions of the study and, therefore, she felt that she did not need to wear the activity tracker anymore.

**Table 1:** Percentage of days with a daily step count above 10,000 and adherence to wearing the Jawbone UP2 during the intervention period.

Participant	Days worn, n (%)^a^	Reasons for not wearing
1	84 (89)	Forgot to wear (4 d^b^); did not wear (last 6 d)
2	77 (91)	Charger did not work (6 d)
3	40 (42)	Forgot to wear (4 d); on holiday (16 d); lost Jawbone UP2 (last 37 d)
4	64 (67)	Forgot to wear (3 d); band broke (last 28 d)
5	87 (99)	Perfect adherence
6	41 (35)	Did not wear (first 6 d); on holiday (9 d); flu (9 d); overall low adherence (51 d)
7^c^	—^d^	Dropout
8^e^	—	No Jawbone UP2 data available (no smartphone)
9	70 (95)	Forgot to wear after charging (3 d)
10	102 (97)	Did not wear on 3 d

^a^The intervention period was extended for some participants due to planned vacations, physical symptoms, family issues and time constraints.

^b^d: days.

^c^Woman no. 7 stopped participating in the trial after 3 weeks.

^d^Not applicable.

^e^Woman no. 8 used the Jawbone UP2, but had no smartphone and, therefore, synchronizing the data was not possible.

### Change in Daily Step Count, Physical Activity Level, and Peak Oxygen Uptake

**Figure 1:**
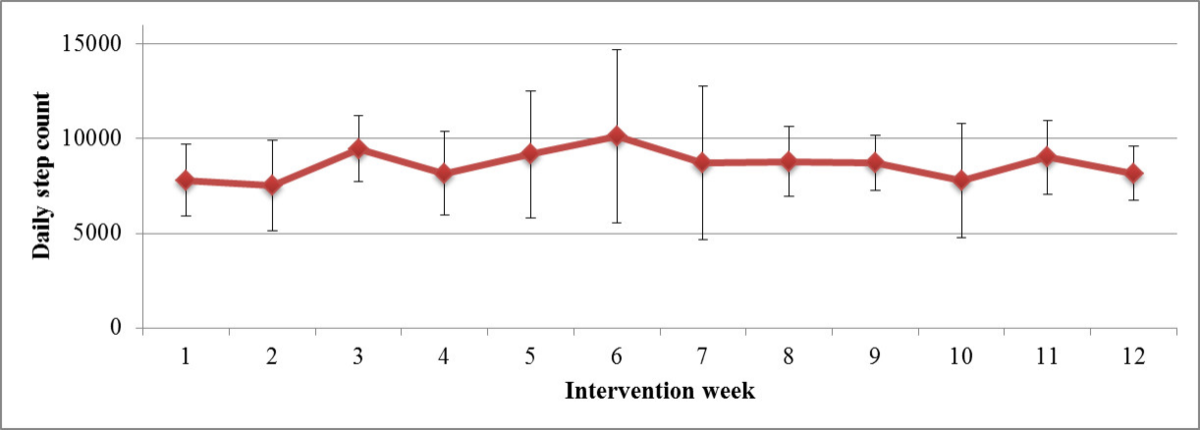
Mean number of steps per day as measured with the Jawbone UP2.

Overall, the mean number of steps per day was 8403 (SD 1994; Multimedia Appendix 1; Figure 1), with the highest step count at week 6 with an average 10,123 steps per day. On an average, patients reached on 30% (SD 20) of the days a step count of more than 10,000 steps. Moreover, 3 of the 8 patients had a mean daily step count of around 10,000, defined as the daily step goal, and 4 patients had an average daily step count between 7000 and 8000.

Mean change from baseline to postintervention in self-reported total physical activity level (including commuting activities, walking, cycling, and sport activities extracted from SQUASH) was plus 79 min per week (SD 123; Table 2), and mean change in VO_2peak_ was plus 1 ml/kg/min (SD 2).

**Table 2:** Physical activity level and peak oxygen uptake (VO_2peak_) at baseline and change during the intervention period.

Participant	Self-reported total minutes of activity; baseline, minutes per week^a^	Self-reported total minutes of activity; change, minutes per week^a^	VO_2peak_; baseline, ml/kg/min	VO_2peak_; change, ml/kg/min
1	60	+90	22	+2
2	360	−210	30	−1
3	330	+110	29	+3
4	30	+90	21	0
5	0	0	23	−2
6	0	+135	19	+4
7^b^	330	—^c^	29	—
8	0	+180	19	+3
9	160	+200	29	+1
10	0	+120	25	+1
Total, mean (SD)	127 (155)	+79 (123)	25 (4)	+1 (2)
Median	45	+110	24	+1

^a^Total minutes of activity including commuting activities, walking, cycling, and sport activities extracted from SQUASH.

^b^Woman no. 7 stopped participating in the trial after 3 weeks.

^c^Not applicable.

**Overview of the themes and subthemes.**

1. The use of an activity tracker and accompanying app raises lifestyle awareness.
  - Activity tracker functions motivate (especially goals and idle alert).
  - More awareness of lifestyle: more physically active and less sedentary time.
2. Patients need personalized advice.
  - Generated advice (Smart Coach) is not applicable to personal situations.
  - Lack of personal advice.
3. Patients need a more realistic total daily physical activity representation.
  - Step goal was too one-dimensional.
  - Prefer the possibility of tracking other physical activity goals.
4. Patients need more integration between the intervention components of the study.
  - No feedback about the activity tracker from the physiotherapist.
  - The activity tracker does not adequately measure fitness activities during supervised exercise sessions.

### Experiences With the Activity Tracker and Accompanying App

Following the thematic analysis, 4 themes emerged from the data (Textbox 2).

#### Theme 1: The Activity Tracker and Accompanying App Raises Lifestyle Awareness

All the women emphasized the important role the activity tracker played as a motivator and tool for gaining insights into their physical activity lifestyles. Woman no. 9 mentioned:

[I] became more aware, the switch is flipped: sometimes I sat for too long a period of time, so I should start moving again. So, it gave me insights.

The main motivating feature of the activity tracker and app was the daily step goal. Almost all the women did something extra to achieve this goal, as woman no. 5 illustrates:

It became an obsession to reach the 10,000 steps.

As an example of this motivation, woman no. 9 said:

You’ve got a goal of a certain number of steps a day and when you’ve almost reached it, you think: “I’ll walk the dog tonight.”

Another motivator for raising awareness was the idle alert function. This function not only motivated the women to start moving before or at the alert, but also made them aware that they should avoid prolonged sitting periods. Woman no. 5 mentioned:

I tried to avoid the [idle alert] notification.

This illustrates how the alert raised awareness to avoid long periods of sedentary time. Woman no. 10 also kept track of her sedentary time so she would move again before the alert vibrated. Woman no. 9 explained how the alert made her conscious of her sedentary behavior:

Oh, it [the activity tracker] is vibrating again, I have to move.

Although the women took different approaches to dealing with the alert, it helped them interrupt their sedentary time, thus raising awareness about their lifestyle.

At the moment of the interview, most of the women were still more aware of their lifestyles, even though they no longer wore the activity tracker for some time. The activity tracker was an important component of the intervention in developing this awareness. Woman no. 9 explained:

It’s something that can help you when you want to live healthier [...] It’s a very good tool to start with. I’d say it increases your awareness and it works very well.

When the women no longer had the activity trackers, they actively tried to maintain a more active and less sedentary lifestyle. Woman no. 8 gave an example:

Now, I more often think I have to get up after an hour, I have to walk.

Woman no. 9 also pointed out:

The funny thing is that I still do it. If a colleague asks: “Shall I get you coffee?” I think: “No, I’ll get it myself.” You become aware that you do not move so much. That’s what you discover in those three months [of the trial].

Comparing her life before and after the trial, woman no. 5 said:

Previously, it was maybe 10 minutes, walking the dog, but now, I walk the dog 20 [minutes] or half an hour more or so, a few times a day.

#### Theme 2: Patients Need Personalized Advice

Almost all the women emphasized a preference for more personalized advice than they received from the Smart Coach. They only viewed directly applicable messages as motivating. Personalized messages about the women’s daily progress toward their individual goals were the most motivating, as exemplified by woman no. 5:

When, for example, in the evening, the device told me: “Well you’ve done a good job, but you still need a certain number of steps, something like 900 or 1,000 steps. Go for a short walk or something else.” And then I went again, or I took the stairs ten times up and down, or I walked an extra time in my garden.

In addition to the motivational aspect, the women enjoyed the personalization of these messages. The longer a woman used the activity tracker, the more personalized messages were generated, and the more she enjoyed them. Woman no. 9 said:

Yes, the Smart Coach, yes, very amusing. The longer you wear it, it figured out how you score, what you like [...]. Then it indicated things that made you think: “It gives me that little bit extra. Yes, I really like it.”

However, the Smart Coach did not always deliver personalized advice. Woman no. 7 explained that the Smart Coach was programmed with standard advice, focusing on healthy people:

If you don’t reach the targets, this Smart Coach will not ask empathetically why it not works [limitations caused by cancer and its treatment]. The only thing he [the Smart Coach] says is “Don’t be a fool and do it. [...] That gives a negative impression.”

She found this very annoying as her disease and its treatment had limited her physical condition and ability to achieve those goals. Nonpersonalized advice was either ignored or experienced as irritating, as woman no. 5 emphasized:

Sometimes it was advice, which I found useful, but sometimes I skipped or ignored it.

Woman no. 9 pointed out that a certain capacity for placing the advice in perspective was needed to cope with the Smart Coach’s messages that did not fit her personal situation. Some women even lowered their goals in the app, so the Smart Coach would not complain; this made them feel better.

#### Theme 3: Patients Need a More Realistic Total Daily Physical Activity Representation

Many women indicated that a disadvantage of the activity tracker was that it only registered steps; physical activities such as resistance exercise and cycling were not registered correctly. The women were trying to develop an active lifestyle of being moderate to highly physically active for at least 30 min a day, but the tracker merely measured their steps and not all types of physical activity. Woman no. 2 said:

Yes, those other activities apart from the steps weren’t registered.

Women no. 7 also pointed out that the activity tracker did not track activities other than walking:

This Smart Coach [...] doesn't add up activities, it only counts steps. For example, I cycled for three hours but I only reached 6000 steps. [...] At that point, it doesn’t say: “You already cycled for three hours.” Instead, it says: “You need 18 more minutes walking the stairs.

The women mentioned 3 activities that they found important and thought should have been captured by the activity tracker and incorporated into their total physical activity representation. First, almost all of them indicated that the fitness workouts at the physiotherapist were inadequately measured. Second, all the women indicated that the bicycle was important as a means of physically active transportation. They were bothered that it was not adequately captured. Finally, swimming was not registered by the activity tracker because it was not water resistant. Woman no. 3 mentioned:

I also found it a pity [...] because at that time I swam an hour and a half every week and I would have preferred to be able to wear it [the activity tracker].

The women were not only bothered that their other activities were not tracked by the activity tracker, but also that they were not specifically shown in the app, as woman no. 1 described while discussing the disadvantages:

And I also can’t find my fitness sessions in the app [...] you can’t find cycling and [lifting] weights [in the app].

Woman no. 5 also shared her frustration about this:

When I went to the physiotherapist for an hour, I had to register it [...] but it didn't show up [the registered activity].

#### Theme 4: Patients Need More Integration Between the Intervention Components of the Study

Almost all the women indicated that they felt a lack of connection between the use of the activity tracker and the accompanying app, and their physiotherapy sessions. Woman no. 3 said:

I did nothing with the bracelet at the physiotherapist sessions and neither did they.

The 2 components were closely related for the women, as the activity tracker measured activity outside their physiotherapy appointments, which was their checkup and feedback moments regarding their physical condition. Woman no. 3 explained:

I thought of it as a feedback moment [...] I’d have some questions about the activity tracker, but I couldn’t ask them to the physiotherapist. It surprised me.

Woman no. 1 noticed*:*

There were supervised sessions in the UMBRELLA Fit program, but the UP [app] was not integrated in what the physiotherapist did [during the sessions].

As the physiotherapy sessions were an important aspect of the trial, the women expected the activity tracker to register activities during these sessions. Woman no. 3 said:

It’d be amazing if everything I did regarding physical activities was registered, including workouts at the physiotherapy sessions.

## Discussion

### Principal Findings

A physically active lifestyle is known to be important for patients recovering from breast cancer, as it may ameliorate the negative side effects of treatment and help to limit some of the comorbidities (eg, heart disease, diabetes, or other cancers) [8,9,25]. This qualitative study investigated the experiences of patients with breast cancer who used an activity tracker and the accompanying smartphone app aimed at increasing physical activity and decreasing sedentary time. The activity tracker was added to a supervised exercise intervention for women who had completed their breast cancer treatment. Aside from logistical reasons for not using the activity tracker, adherence was high. Daily step count, self-reported physical activity level, and VO_2peak_ of participants slightly increased following the exercise intervention. As the activity tracker was part of the exercise intervention, it was not possible to draw conclusions on the isolated effect of the activity tracker and associated app, but a recent meta-analysis indicated that exercise interventions comprising activity trackers and smartphone apps were more effective than exercise interventions without activity trackers and smartphone apps [26].

From our interviews with 10 participants, 4 important themes emerged: (1) the use of an activity tracker and accompanying app raises lifestyle awareness, (2) patients need personalized advice, (3) patients need a more realistic total daily physical activity representation, and (4) patients need more integration between the intervention components of the study.

From the interviews, we found that the activity tracker and accompanying app functioned as a motivational tool and created more awareness of physical activity behavior and sedentary behavior. The women indicated that the daily step goal defined in the accompanying app was the most important motivator. Similar results were found in a comparable study of patients with breast cancer by Nguyen et al [17]. They found that goals presented in an achievable and easy-to-understand indicator of physical activity (eg, step goal presented in numbers) worked well. It helped the patients with breast cancer to be more aware of their physical activity levels and to incorporate physical activity into their daily routines. This is also supported by Wang et al [27], who reported that, in general, using a physical activity app facilitated more exercise. Furthermore, feedback concerning the progress toward their goal made the women more determined to stick to their daily activity goal which was also in line with Nelson et al [10].

The next theme that emerged from the interviews was a need for more tailored and personalized advice. The women indicated that the Smart Coach messages which were applicable to their activity levels were motivating and enjoyable. The advice given in a positive way encouraged the women to be more engaged in an active lifestyle. These kinds of positive encouragements were comparable with the rise in motivation to reach goals, when rewards were provided [28]. However, some of the messages generated by the Smart Coach did not fit the women’s situations and were tailored to activity levels of the general population. As all the women had undergone cancer treatment and experienced disease- and treatment-related side effects as fatigue and a decreased physical fitness level, it was sometimes frustrating to read messages generated by a system that did not consider the side effects of the treatment. Our results suggested that using generic commercial trackers and their associated software for purposes of rehabilitation support may require additional design adaptations and references that are specific to the person and the limitations imposed by the disease and treatment. Women also mentioned the important role the physiotherapist played as a physical activity expert who was very helpful in the process of becoming fit. Therefore, combining the physiotherapist’s feedback with the immediate, quantitative feedback from the tracker in the app was seen as a potential improvement. In addition to messages, the physiotherapist could give personal advice about matters such as the women’s goals and possible adjustments to them.

Other studies support this suggestion of combining activity tracker data with feedback from a medical professional [25,29,30]. Including input from a health professional could make interventions more effective. Nguyen et al [17] also suggested that support from peers with similar conditions may be helpful both in terms of practical advice (eg, what is a realistic goal to strive for) as well as psychosocial support, making the intervention more enjoyable and motivating. This is in line with recent insights related to personal tracking, which increasingly acknowledge the importance of data sharing and social interactions around data [31]. The third theme emerging from the interviews was the need for a more realistic total daily physical activity representation. As the activity tracker was not capable of accurately measuring forms of physical activity besides steps, these activities were also not part of the daily amount of physical activity shown in the app. The most important inadequately captured activity was cycling, which is a common means of transportation in the Netherlands. Dutch women cycle around 2.3 kilometers every day [32]. As cycling is so prominent in the Netherlands and contributes significantly to an active lifestyle, not registering this activity gives an inaccurate impression of a person’s physical activity level. This may not be a problem in cultures where cycling is not that prominent. In addition, the women indicated that swimming and fitness sessions were important physical activities.

As the representation of total physical activity level is inaccurate, it is more difficult to monitor their path to a physically active lifestyle. This might lead to frustration and reduce motivation to continue using the activity tracker. Rosenburg et al [18] also reported inaccurate measuring of other activities apart from walking as a barrier to use an activity tracker in men with prostate cancer. Thus, additional design requirements for activity trackers in this context include a greater diversity in automated activity registration, as well as water resistance. This allows women to swim and bath with the tracker and have a more accurate activity representation that includes more activities. Activity trackers with these features are now widely available and should be considered for future studies.

The women also liked the feedback system (ie, Smart Coach) in the accompanying app. An opportunity for improvement could be to use an activity tracker with an integrated screen that gives immediate feedback, thus eliminating the need to go online to inspect the information in the app. However, this would be more expensive than the current system.

The last theme that emerged from the interviews was the need for more integration between the health professional and the activity tracker. As discussed above, a health professional can add value to the patient’s intervention experience when provided with data from the activity tracker (eg, the health professional could help the patient determine an appropriate higher or lower step goal and give her specific tips for reaching the goal). In addition, the health professional could fulfill the role of the “Smart Coach” in which the positive qualitative feedback could be adjusted to individual patients.

### Strengths and Limitations

This study had several strengths. First, it was a field study where participants used the activity tracker during a 12-week intervention program. This allowed them to get beyond first impressions and realistically use and evaluate the activity tracker in context. Second, through in-depth interviews, we gained insight into the use of, and experiences with, the activity tracker, the accompanying app, and its functions (eg, the idle alert and the Smart Coach) in contrast to reviews focused on the use of an activity tracker alone. This gave insight into the drivers and barriers to acceptance and into the use of activity trackers for patients with breast cancer. These interviews helped us identify several important requirements and improvements for next-generation trackers to be used in the context of patient recovery. Third, as this study was part of a larger medically supervised intervention, we were able to explore some of the strengths of combining activity tracker feedback with tailored and personalized feedback from medical professionals. This pertains to the larger issue of patient empowerment through self-tracking and the changing roles of patients and doctors, as digital technology increasingly democratizes through digital technology. Finally, because most activity trackers and smartphone apps have comparable functions (eg, step counting, goal setting and tracking, a daily report, an idle alert, and sleep tracking), our results also apply to other activity trackers apart from the Jawbone UP2.

A study of this kind also has limitations. First, the particularities of the activity tracker we used may not generalize to all trackers, and new technological developments in sensing, data processing, and interface design will allow current and new-generation trackers to overcome some of the issues we identified in this study. Second, although many of our insights are likely to hold true for populations other than recovering patients with breast cancer, our results also illustrate that the particularities of a disease and the effects (or side effects) of its treatment are important. This supports a general argument favoring personalized approaches through adaptive digital interfaces and personal coaching over a one-size-fits-all approach. By necessity, this also limits the generalizability of our findings to other specific patient populations. Finally, even though our study participants used the tracker for several weeks, it remains to be seen what the long-term health effects could be of using an activity tracker in comparison with other fitness-promoting programs. Also, the key factors contributing to long-term use and effectiveness of activity trackers are a topic of interest for future investigation.

### Conclusions

This study explored experiences with an activity tracker and its usage in an exercise intervention among inactive patients with breast cancer. The interviews showed that an activity tracker raises awareness of a physically active lifestyle and sedentary behavior. Our study also showed the potential of using a wearable activity tracker to improve supportive care after primary treatment for breast cancer. However, there is a need for a more realistic representation of the total daily physical activity, a more personalized advice that is tailored to their current situation after breast cancer treatment, and a better integration of the activity tracker into clinical practice. To optimize the use of an activity tracker in clinical practice, we suggest to base personalized advice on references that are specific to the patient population and to integrate the use of activity trackers and smartphone apps in rehabilitation programs, which requires more intensive guidance of a health care professional on usage and goal setting.

## Appendix

Multimedia Appendix 1 Mean number of steps per day per week during the intervention period.

## Abbreviations

SQUASH: Short QUestionnaire to ASsess Health-enhancing physical activity
UMCU: University Medical Center Utrecht
VO2peak: peak oxygen uptake
