# Supplementary material for: Breast Cancer Survivors’ Experiences With an Activity Tracker Integrated Into a Supervised Exercise Program: Qualitative Study
Source: JMIR Mhealth Uhealth. 2019 Feb 21;7(2):e10820. doi: 10.2196/10820 (PMC6403530; doi:10.2196/10820)
Supplement: Multimedia Appendix 1 [file mhealth_v7i2e10820_app1.pdf]

Multimedia Appendix 1. Mean number of steps per day per week during the intervention period.

	Mean number of steps per day (per week)												
Participant	1	2	3	4	5	6	7	8	9	10	11	12	Total
1	6,959	8,248	6,365	6,974	6,415	8,626	6,928	7,349	8,380	5,960	6,095	6,123	7,035
2	9,285	7,618	10,214	8,880	15,157	19,603	12,626	12,463	6,654	NA	10,926	8,475	11,082
3	7,867	6,088	9,537	NA	NA	5,824	6,354	7,336	7,297	NA	NA	NA	7,186
4	7,552	9,335	12,058	10,039	9,725	13,698	16,568	9,151	9,696	11,596	NA	NA	10,942
5	10,657	10,933	10,935	9,903	11,121	9,221	7,664	8,263	8,269	9,874	10,422	9,456	9,727
6	4,225	4,229	9,536	3,873	4,805	5,652	3,481	NA	NA	NA	NA	NA	5,114
7 [b]	NA	NA	NA	.	.	.	.	.	.	.	.	.	
8 [b]	NA	NA	NA	NA	NA	NA	NA	NA	NA	NA	NA	NA	
9	7,125	4,545	8,437	7,738	8,569	9,198	7,990	7,393	9,891	7,408	9,532	.	7,984
10	8,667	9,190	8,594	9,641	8,365	9,168	8,020	9,554	10,657	4,083	8,070	8,587	8,550
Total Mean (SD)	7,792 (SD 1,895)	7,523 (SD 2,391)	9,459 (SD 1,727)	8,150 (SD 2,208)	9,165 (SD 3,357)	10,123 (SD 4,566)	8,704 (SD 4,057)	8,787 (SD 1,854)	8,692 (SD 1,454)	7,784 (SD 3,003)	9,009 (SD 1,957)	8,160 (SD 1,427)	8,403 (SD 1,994)

d days; *NA* not available; *SD* standard deviation; . = not within intervention period

[a] Only weeks with information available of daily step count on three or more days were taken into account.

[b] Woman no. 7 stopped participating in the trial after 3 weeks. Woman no. 8 used the Jawbone UP2, but had no smartphone and, therefore, synchronizing the data was not possible.
